# Idiopathic Intractable Diarrhoea Leading to Torsade de Pointes

**DOI:** 10.1155/2016/3845108

**Published:** 2016-05-23

**Authors:** Kyriacos Mouyis, Darlington Okonko, Constantinos G. Missouris

**Affiliations:** ^1^Frimley Health NHS Foundation Trust, Wexham Park Hospital, Cardiology Department, Slough SL2 4HL, UK; ^2^King's College London BHF Centre, King's College Hospital, London SE1 7EH, UK

## Abstract

An 81-year-old lady was admitted to our hospital with a 3-year history of noninfective diarrhoea and recurrent syncopal events over the last 3 months. Her initial electrocardiogram (ECG) revealed trigeminy and prolonged QTc interval. She had a structurally normal heart with no coronary artery disease. Investigations revealed low potassium at 3.0 mmol/L. Sigmoidoscopy and colonoscopy suggested a possible diagnosis of diverticulitis. Soon after admission she had an unresponsive episode with spontaneous recovery. Telemetry and Holter analysis confirmed multiple episodes of polymorphic ventricular tachycardia (Torsade de Pointes). Following electrolyte supplementation the episodes of polymorphic VT improved. Due to the protracted nature of the diarrhoea, the recurrent syncopal events, and recurrent hypokalaemia documented over recent years, an Implantable Cardioverter Defibrillator (ICD) was sanctioned by the multidisciplinary team (MDT). In summary, chronic diarrhoea may result in life threatening polymorphic VT due to hypokalaemia and QTc prolongation. In these patients an ICD may be considered.

## 1. Introduction

Electrolyte abnormalities are common in patients with prolonged diarrhoea. We report the case of a lady, the first we are aware of, who suffered from hypokalaemia and a history of recurrent syncopal events related to Torsades de Pointes as a result of persistent and intractable diarrhoea.

## 2. Case Presentation

An 81-year-old lady was admitted to our hospital with recurrent falls associated with brief loss of consciousness. She denied any other cardiac symptoms. Her past medical history included an embolic cerebrovascular event a year earlier with no residual focal neurology, hypertension, treated hypothyroidism, diverticulosis, anaemia of chronic disease, and chronic noninfective diarrhoea of 3 years' duration. This had been extensively investigated by several gastroenterologists and colorectal surgeons.

On admission she was passing 8–10 loose motions a day. She was on treatment with levothyroxine 125 micrograms OD, clopidogrel 75 mg OD, aspirin 75 mg OD, atorvastatin 40 mg OD, ranitidine 150 mg BD, zopiclone 7.5 mg OD, citalopram 10 mg OD, folic acid 5 mg OD, loperamide 2 mg TDS, co-codamol 8/500 2 tablets QDS, and desloratadine 5 mg OD.

On examination she was apyrexial, the pulse was 60 beats per minute and regular, and the supine blood pressure was 157/55 mmHg. Saturation on air was 95%. The rest of the cardiovascular, respiratory, and abdominal examination was within normal limits. There were no history of liquorice use, no clinical evidence of somatic neuropathy, and no evidence of adrenal adenomas on abdominal CT examination done prior to admission.

**Figure 1:**
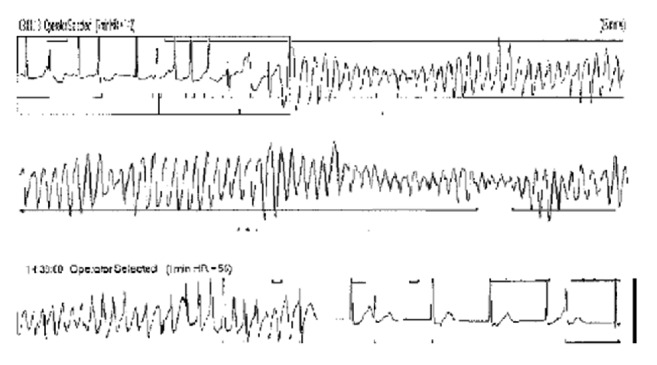
Rhythm strip recorded during the episodes of syncope showing polymorphic VT, preceded by sinus rhythm with atrial ectopic beats.

The investigations were as follows: haemoglobin 12.7 g/L, sodium 143 mmol/L, potassium 3.0 mmol/L, chloride 101 mmol/L, bicarbonate 27.6 mmol/L, pH 7.46, magnesium 0.72 mmol/L, adjusted calcium 2.21 mmol/L, phosphate 1.08 mmol/L, TSH 0.22 *μ*/mL, free T4 25 pmol/L, and C Reactive Protein (CRP) 89 mg/L. The admission resting ECG revealed ventricular bigeminy and prolongation of the QTc at 519 msec (normal for females < 470 msec), measured using Bazett's formula as heart rate was less than 65 bpm. Telemetry and Holter analysis confirmed the presence of multiple episodes of polymorphic VT (Torsades de Pointes) (Figure 1). An echocardiogram confirmed a structurally normal heart with a normal ejection fraction of greater than 55%. Coronary angiography confirmed normal appearances with no evidence of overt coronary artery disease.

**Figure 2:**
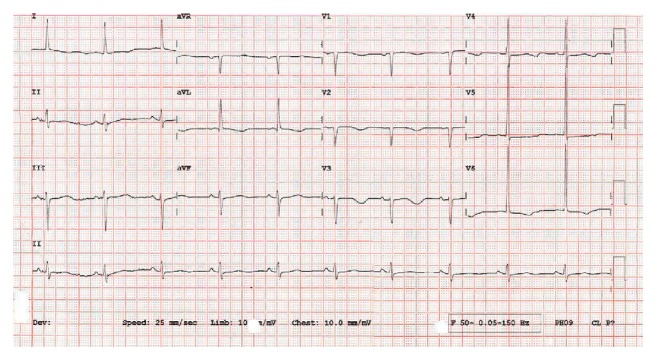
12-lead ECG recorded after termination of episodes of TdP, showing prolongation of the QTc interval.

The patient's episodes of polymorphic VT stopped following administration of intravenous and oral potassium as well as magnesium supplementation (Figure 2) and the discontinuation of citalopram, which has been associated with development of long QT syndrome and Torsade de Pointes as an adverse drug reaction [1]. The QTc normalised within 48 hours following this. The patient was treated with loperamide 4 mg prn and amiloride 5 mg daily to correct the hypokalaemia. Following these interventions potassium levels remained above 4.0 mmol/L. Despite the administration of amiloride and normalisation of the potassium the patient was still experiencing ongoing diarrhoea. Additionally there were past blood tests (ordered by the patient's GP) documenting low potassium levels in the preceding 2 years. With that in mind the cardiology MDT felt that the precipitating cause of the Torsades de Pointes was not fully addressed and thus an ICD would offer the best change of preventing an arrhythmic death. An ICD (dual chamber, Medtronic Evera) was thus prophylactically implanted. Due to the patient's age and in the absence of any previous or family history of syncope and ventricular arrhythmias no genetic tests were performed.

On follow-up after six months, she remained asymptomatic with no further syncopal events or symptoms of light headedness, blurred vision, or feeling faint. Repeat blood tests by her GP at 3 and 6 months showed potassium of 4.1 and 4.2 mmol/L, respectively, and she was continuing to take amiloride. No arrhythmias were detected on interrogation of the ICD at the follow-up.

## 3. Discussion

Polymorphic VT (Torsade de Pointes) is a distinctive form of ventricular tachycardia characterised by oscillation of the QRS complex around the isoelectric axis and it predisposes to sudden cardiac death. QT interval prolongation is a prerequisite for this arrhythmia. Prolonged repolarisation is mediated via several subtypes of sodium and potassium channels in cardiac myocytes, mainly by inhibiting the rapid component of the delayed rectifier potassium current (*I*_Kr_) [2].

Conditions that cause a prolonged QT interval such as congenital syndromes (e.g., Jervell and Lange Nielsen, Romano Ward) predispose to polymorphic VT. Women also have an inherently longer QT interval due to intrinsic sex differences in the cardiac conductive tissue and so are more at risk. Furthermore, electrolyte abnormalities, and in particular hypokalaemia (but also hypomagnesemia and hypocalcaemia), predispose to the above arrhythmia by increasing the length of delayed repolarisation and consequently the QT interval [2]. Possible precipitants for hypokalaemia include drugs (e.g., diuretics such as indapamide, amphotericin B, aminoglycosides, and psychotropic medications), excessive GI tract losses (e.g., diarrhoea, vomiting), or renal losses (e.g., renal artery stenosis, primary hyperaldosteronism) [2].

A small number of case reports have been published establishing an association between diarrhoea-induced hypokalaemia and polymorphic VT. In one report hypokalaemia was precipitated by the use of the antipsychotic drug thioridazine and in another by the use of laxatives [3, 4]. In both cases the polymorphic VT was terminated following the withdrawal of the implicating drug. In another study Kusano et al. reported nonsustained VT with normal QTc interval in the context of hypokalaemia and diarrhoea [5]. Krahn et al. reported a patient with hypokalaemia secondary to inadvertent laxative abuse who required cardioversion for Torsades de Pointes [4].

In our case the patient had more than one reason for prolonged QT and subsequent Torsades de Pointes. We believe that the temporal relationship of events offers some support to our assertion of hypokalaemia being the driver of the arrhythmia rather than citalopram use. Citalopram has a half-life of approximately 35 hours and is often prolonged in the elderly. The patient's arrhythmic episodes resolved after less than 48 hours following correction and maintenance of potassium levels at more than 4.0 mmol/L. Additionally there is evidence for a dose related relationship between citalopram and QTc prolongation. This lady was on 10 mg which is a low dose regime; thus, it is less likely that this would have caused a significant QTc prolongation.

Our case report demonstrates that long standing diarrhoea is an important and reversible cause of hypokalaemia and QTc prolongation that can result in life threatening polymorphic VT. In addition, it demonstrates that due to the protracted nature of the diarrhoea and the recurrent syncopal events an Implantable Cardioverter Defibrillator (ICD) was deemed to be of potential benefit. This was because no definitive treatment was found for her diarrhoea which likely precipitated her hypokalaemia and the subsequent ventricular arrhythmia. One could argue that the initiation of amiloride and the discontinuation of citalopram were sufficient to terminate the arrhythmic episodes without the need for an ICD implantation. Nonetheless, the Torsade de Pointes load and the ongoing diarrhoeal episodes were such that the unanimous decision of the cardiology MDT was in favour of implanting an ICD as some of the above life threatening events may occur long after the implantation of the device.

All patients, therefore, presenting with syncope (or symptoms of light headedness, blurred vision, or feeling faint) and long term diarrhoea, should be assessed for hypokalaemia and electrolyte disturbances that may predispose to polymorphic VT. In these patients correction of the above electrolyte abnormalities, with or without ICD implantation, is likely to improve cardiovascular prognosis and outcome.
